# Correction: Beyond eves and cracks: An interdisciplinary study of socio-spatial variation in urban malaria transmission in Ethiopia

**DOI:** 10.1371/journal.pgph.0003593

**Published:** 2024-07-31

**Authors:** Claudia Nieto-Sanchez, Stefanie Dens, Kalkidan Solomon, Asgedom Haile, Yue Yuan, Thomas Hawer, Delenasaw Yewhalaw, Adamu Addissie, Koen Peeters Grietens

The ORCID iD of the second author is incorrect. Please see the author’s correct ORCID iD here:

Author Stefanie Dens’ ORCID iD is 0000-0002-6680-3645 (https://orcid.org/0000-0002-6680-3645).
