# Supplementary material for: Beyond eves and cracks: An interdisciplinary study of socio-spatial variation in urban malaria transmission in Ethiopia
Source: PLOS Glob Public Health. 2022 Apr 22;2(4):e0000173. doi: 10.1371/journal.pgph.0000173 (PMC10021683; doi:10.1371/journal.pgph.0000173)
Supplement: Attachment — Submitted filename: COVER letter PLOS Global Pub Health_rebuttal letter_SENT.docx [file pgph.0000173.s001.docx]

| Department of Public Health  Unit of Socio-Ecological Health Research |  |
| --- | --- |
| 10/12/2021 |  |

Dear Editors,

First of all, we want to express our appreciation for the comments obtained from the reviewers in our first submission. However, we consider that most of the reviewers’ comment were focused on the entomological data of the study, without due attention to the contributions of the interdisciplinary methodology hereby proposed. In the following table we indicate how their comments were addressed in this submission and provide our rationale for those that were not.

We apologize for this late repsonse and will be happy to address any further comment.

Best regards,

Claudia Nieto-Sanchez, PhD

Researcher

Unit of Socio-Ecological Health Research - SEHR

Institute of Tropical Medicine Antwerp

cnieto@itg.be

| **Journal requirements** | |
| --- | --- |
| We note that participants provided oral consent. Please state in the Methods: - Why written consent could not be obtained - Whether the Institutional Review Board (IRB) approved use of oral consent - How oral consent was documented | We acknowledged that this was an oversimplification of our consent process. Consent was provided both orally and in written format by all research participants.  After accepting to participate in the study, participants’ consent was registered in audio recordings. Subsequently, written informed consent was collected. An extended explanation of the consent process is provided in lines 409-415. Procedures were approved both by ITM and AAU IRB. |
| Open access data | Addressed in email to Editor. |
| In your Methods section, please provide additional information regarding the permits you obtained for the work. Please ensure you have included the full name of the authority that approved the field site access and, if no permits were required, a brief statement explaining why | Permissions to access field sites were provided by Jimma’s Major’s office, Jimma University (for the condominium under its administration), and condominium residents before each instance of data collection. |
| **Reviewer 1** | |
| Line27? what progress? 27-28: what vectors? What out breaks? malaria? 31-33: this is not clear aim! what is the integrated methodology? (what is integrated) 31: This sentence is very confusing! 33: what is this unit? be clear 36: This should be the aim! No? 37: what study design was this? 47: abbreviation here is inappropriate! | The abstract has been modified to specify questions posed by the reviewer |
| There is no information on the epidemiology of the disease, the control efforts put and also the NEED or GAP that this research findings will fill! | Information regarding malaria epidemiology is provided in lines 91 to 111. Additional information about malaria in Jimma is provided under *Study Site* in the *Methods* section (lines 190 -202) |
| 88: what is integrated? | We are not referring here to a traditional interdisciplinary study with separate study arms, but to an interdisciplinary study that considers emerging results from different disciplines to guide methodological decisions and analysis during the research process. The phases of integration are described under the *Study Design* subheading, lines 181-183. |
| 90: We hypothesized that……………… this is unclear | In order to reduce any confusion, this sentence was reformulated (lines 114 – 122). |
| 93: I feel there is a need to expand on this as you have put in the abstract about Ethiopia adopting this type of housing. | An extended explanation of the IHDP program is provided under Study Site, lines 209 -276 |
| 112-116: since the aim is malaria transmission interruption, you should give justification for this work hence give a little background as to why these vector control tools are no sufficient for malaria control/elimination. I suggest this to be in the introduction | Additional information has been provided in lines 169 - 178: “Planning for scaling-up malaria prevention and control interventions started in 2003 with the support from the Global Fund to Fight AIDS, Tuberculosis and Malaria (GFATM) and in 2004, the Ethiopian Federal Ministry of Health introduced artemisinin-based combination therapy (ACT) as the first-line drug for treatment of *P. falciparum* malaria, along with rapid diagnostic tests (RDT), to improve diagnosis, and distribution of long-lasting insecticidal nets (LLINs) as main prevention method. Major scale-up began in 2005 with country wide distribution of RDTs, ACTs, LLINs and implementation of indoor residual spraying (IRS) [28]. Currently, Ethiopia has set the goal of malaria elimination by 2030. However, current vector control interventions, mainly LLINs and IRS, are becoming less effective due to insecticide resistance, outdoor transmission, and change in vector biting and resting behavior [29]” |
| 117-120: It is well to be information, but how is this linked to malaria transmission? i mean this can very well be in introduction if it is background information or is it the actual study site? | The methodological approach taken in this case is context-specific and responds to the particular conditions of IHDP sites. Given their relevance for our decision making in relation to the study design, we consider that this information is best placed under the Methods section. |
| Also a map should be added for visual aid | Fig 1 is a map with the localization of the condominiums included in this study. |
| 132-133: so what were the combined study methods? | We think this is suffciently explained in the manuscript. Each discipline involved in the study described methods of data collection and analysis in the 'Methods' section. |
| 134-135: this is vague! what did you join? be specific | The purpose of this sentence is to introduce the process explained in detail by each discipline in the subsequent paragraphs. |
| 143-145: First, when exactly were these collections made? What months? what season? | This is addressed in the MS as "The mosquito collection started in September and continued through October and November which is the major malaria transmission peak season in Ethiopia" (lines 309 -310) |
| The trap nights is confusing, you had a total number of 36 trap nights both indoor and out doors, 12 trap nights in 3 different floors. Then you say a total of 108 traps for 12 trap night??? This is unclear because you have not explained your study site clearly! how many sites? what was the unit of measure? houses? if so, what kind f houses are these? I do not see the need of having a section data collection and then a section of mosquito sampling! combine the two and make give clear description of what was done, if need be, use figures for clarity | The following information has been added in lines 295 -310: “Three IHDP residential sites were included in this study. In each residential sites 3 blocks with ground, first and second floors were randomly selected. One house from each floor was selected for monthly mosquito collection using Centre for Disease Prevention and Control (CDC) traps for 3 months. In each month mosquitoes were collected both indoor and outdoor for two consecutive nights using CDC light traps. Hence, in each month, the CDC LTs were set in three blocks on ground, 1st and 2nd floor for two nights. CDC light traps were set inside selected houses near an occupied bed at a height of 1.5 meter from 18:00 to 06:00 hr in the night to collect indoor host seeking mosquitoes. For the outdoor host-seeking mosquito sampling, CDC light trap was also set in the vicinity (within 8 meter) of sentinel houses from 18:00 to 06:00 hr. Accordingly, mosquitoes were collected for a total of 36 CDC-nights (18 indoor and 18 outdoor) from each site every month for three months which was 54 trap nights indoor and 54 trap nights outdoor per residential site. Thus, a total of 108 trap-nights per residential site and this would give us an overall total of 324 trap-nights for the 3 residential sites for the entire study period. N.B. [3 residential sites*3blocks*3floors*3months*2nights/house*2places of mosquito collection (indoor & outdoor)]. |
| 146: first time, write it in full | Addressed. CDC traps is written as Center for Disease Prevention and Control traps in the body of the revised MS. |
| 152-153: data analysis means explaining how you analyzed your data; parameters analyzed, how they were analyzed (if using software) or formulas, then what are those formulas? if its descriptive data, you say so. Bu this is lacking here, rather your are just giving explanations rather that analysis done! | Data analysis procedures were further explained in lines 338-348: “Data were entered in a spread sheet and analysed using SPSS version 20.0 (SPSS, Inc., Chicago, IL). Mean indoor and outdoor mosquito density was determined using T-tests. Moreover, the mean difference in mosquito density collected from the different floors of the residential blocks and densities of mosquitoes collected in the different months was compared using ANOVA. P. value < 0.05 was considered significant during the analysis” |
| 154: 1st floor was not included? if so, why? Also at the top you had mentioned only ground, 1st and 1nd floors! what changed? | The different floors are corrected as ground, 1st and 2nd floors in the revised MS |
| 155: What were those stages? | We thank the reviewer for his/her critical observation. It is a typo error, the "physiological stage" is now corrected as "physiological state" in the revised MS. |
| 158-159: how was entomological innoculation rate calculated? what formula was used? | The calculation of EIR is removed from data analysis section in the revised MS as we did not determine the sporozoite rate due to low number of Anopheles mosquitoes collected |
| 160: what do you mean? also, before abbreviating anything, you need to write it in full | Addressed as CDC traps is written as "Center for Disease Prevention and Control" traps in the body of the MS . |
| 162-164: need to list the variables that were collected | At the beginning of the ‘Data collection’ section, under Medical Anthropology, we state that data on “uses and appropriations of the space, processes and structures of social organization, and factors influencing the experience of residency in IHDP condominium housing (i.e., homes and communal spaces)” were collected. Rather than discrete variables (variables are part of the quantitative research paradigm; themes are part of qualitative analysis), these large themes guided instances of data collection and analysis under the methodologies described in the manuscript. |
| 179: how? | A clarification clause was added to make the analysis process more explicit (line 386) |
| 181: what were the variables? | Rather than variables (variables are part of the quantitative research paradigm; themes are part of qualitative analysis), themes guide data collection and analysis in qualitative research. Analytical categories emerging from the analysis phase are introduced the results section (lines 301 to 305). |
| 200: Author to provide details such as the ethics approval reference number | Ethical approval: Addis Ababa University, College of Health Sciences, IRB; Date of approval: 29/10/2019; Approval reference number: 081/19/2019 |
| 205: on average per night, how many mosquitoes were collected? in the assumption tat your targeting malaria vectors, i think this would have been important to focus on Anopheles. | Yes, in our study we targeted Anopheles mosquitoes but we didn't run test of significance as there was a low number of (n =34) Anopheles mosquitoes collected (maybe due to the use of aerosols by households to control household pests). Therefore, as culicine mosquitoes also create nuisance to people, we pooled both Anopheline and Culicine mosquito data to determine mean mosquito density per trap night and the result is provided in lines 418 – 423. |
| In the methodology section there was mention of physiological status, where are the results? | Of the total Anopheles mosquitoes 33 (91%) were unfed and 3 (9%) were fed (lines 424 - 425). |
| 206: all those trap nights? when were these collections made? what season? | The mosquito collection started in September and continued through October and November which is the major peak malaria season in Ethiopia. |
| 207-209: This a scientific paper suppose to have results that are scientifically sound, therefore, the need to have statistical significance in your results is paramount. | We ran tests of significance to compare the difference in mosquito density between indoor and outdoor, among different floors and months of mosquito collection and the results of the analysis are included in the revised MS. |
| 210-211: this is percentage... how about mean per collection night per position of trap (indoor vs outdoor)? | The mean density of combined anopheline and culicine mosquitoes was 8.5mosquitoes/trap-night, with significantly higher outdoor density t =8.1, p < 0.001. Moreover, the mean density of the mosquitoes collected from the ground, first and second floor of the residential blocks was 13.4, 7.0 and 5.2 mosquitoes/trap-night, respectively (lines 427 -472). |
| 211-216: All these are just statements with proportions but with nos statistics to show the significance | *Response*: We ran t-tests to assess whether there was significant difference between mean indoor and outdoor mosquito density , we also used ANOVA to compare differences in mean density of mosquitoes collected from different floors and months. |
| 217-218: how significant was the difference between floors and between species? | Lines 269 – 270 state: “There was a significant difference in mean density of mosquitoes collected from the different floors of the blocks [F(2,321)=32.18, p < 0.001]. Pairwise comparison of the mosquito density from the different floor levels showed that highest mean mosquito density was obtained in the ground floor (p < 0.001).” |
| 219-220: how significant was the difference between floors and between species? | Please see the above response. |
| 450-451: what species? | The primary and secondary malaria vectors recorded in the sites were *Anopheles arabiensis* and *An. pharoensis*, respectively and this is included in the revised MS (Lines 424 - 425). |
| 453-456: Is the hypothesis proven? if so, then this needs to be said out right and by how much has it been proven (ie statistics) | The initial paragraph of the Discussion section was rewritten to reflect changes in the Introduction section. It now states ‘Residents of the observed condominiums were at risk of malaria infection due to the occurrence of both the principal (*An. arabiensis*) and secondary malaria vector (*An. pharoensis*) across sites, as confirmed by the presence of fed mosquitoes collected from indoor and outdoor areas. Although homes lay-out and construction practices remained important to explain mosquito abundance within the home space, extending the unit of research from individual homes to the settlement and its urban context, facilitated identification of systemic interactions that could sustain transmission even in outdoor settings. Systemic interactions creating variations in mosquito abundance and distribution in this study were mainly related to building practices that ignore the logics of territory (‘genius loci’) and its context, deficiency of water/sewerage and waste disposal management systems, and adaptations of the space derived from the heterogeneous demands of residents.” |
| 462-464: no likely hood ration was calculated, hence how can you make such assumption? Need to calculate this ration for your statement to be true | As analysis of mean comparison using ANOVA showed significantly higher mosquito density in the ground floor compared to other floors, individuals living in the ground floor of the residential blocks are likely to be at higher risk of mosquito bites. |
| 464-465: in the FGD and interviews, was this mentioned? or in the study sites analysis, were these attributed mentioned or collected as part of the study? if so, then it should be stated here and in results, if not, then look for supporting references | We are providing all data (entomological, ethnographic and architectural) as evidence for this claim. |
| 466-468: these are assumptions not proven by facts! either you have collected this data or you reference existing studies that have reported this. | We are providing all data (entomological, ethnographic and architectural) as evidence for this claim. |
| 469-470: Is this the first study to show the relationship between residential homes and proximity to mosquito breeding sites? | Multiple studies (included as references) have established relationships between different features of home structures and malaria transmission. The references provide an explanation for the contextual factors identified in this study. |
| 474-475: have this been reported elsewhere? | Not to our knowledge. |
| 495-496: In the methodology, you did not specify where these traps were put both indoors and outdoors! you need to give these details | CDC light traps were set inside selected houses near an occupied bed at a height of 1.5 meter from 18:00 to 06:00 hr in the night to collect indoor host seeking mosquitoes. For the outdoor host-seeking mosquito sampling, CDC light trap was also set in the vicinity (within 8 meter) of sentinel houses from 18:00 to 06:00 hr (lines 300 -303) |
| 537-540: As pointed at the limitation of the study, is this truly demonstrated? | The study builds a case for the consideration of systemic interaction at the level of the home, settlement and landscape for malaria transmission. |
| 540-541: I doubt this has been effectively demonstrated by this study, i would say it calls for further research | Although more research is deemed necessary, our results demonstrate that transmission dynamics cannot be studied only at the level of the home space, without further consideration of systemic interactions at settlement level. |
| **Reviewer 2** | |
| The statements “Success in vector has been recently…” is not clear and needs to be revised for clarity and understanding. | This sentence was removed. |
| The introduction provided by the authors is satisfactory. However, the authors could state clearly previous policies/interventions carried out in Ethiopia to mitigate malaria transmission in the country, especially in the urban cities, and how their current study will supplement those efforts in this regard. Part of this was rather noted in the methods section instead of in the introduction. | The introduction has been reworked. Information requested by the reviewer is now included in lines 117 -178 |
| What informed the authors choice of September to November for the data collection (fieldwork)? Could it be that it was the rainy or dry season or both? This should be explained. | The reason for choosing the months of September to November was to accommodate mosquito density and infection during the high malaria transmission season. |
| What informed the authors selection of 3 sites, 36 trap-nights, and data collection over a period of 3 months? This warrants brief explanation. | Explanation has been provided in lines 309 -310 |
| Authors should provide citation for the NVIVO software in line 182 at page 9. | Citation included |
| In line 215-216 at page 10, the statement “In the observed communal residential apartments risk of outdoor mosquito bite was higher than indoor” cannot be supported by the data as no such analysis was done to estimate and compare the risk of mosquito bite by indoor or outdoor status. The statement “…risk of outdoor mosquito bite was higher than indoor …” has a statistical meaning or the authors are just using it loosely? Thus, this statement should be removed or rephrased to reflect the kind of analysis done. Similar statements in other parts of the manuscript, including the discussion section should be rephrased. | Results of the analysis using T-test to assess differences in mean indoor and outdoor mosquito density showed that there was significantly higher mean outdoor than indoor mosquito density (t =8.1, p < 0.001). Therefore, inhabitants are likely at higher risk of mosquito bites outdoor than indoor. |
| Again, statements like “entomological results showed that inhabitants residing in ground floors were more likely to be at risk of mosquito bites when compared to those residing in the first and second floor” in lines 462-464 at page 20 cannot be supported with the present data presented because no such formal analysis was done and presented. Could the authors show where they estimated such measures (e.g., odds ratios or relative risk or other similar measures) or they are using the terms like “… more likely to be at risk of mosquito bites …” loosely. Similar statements should be rephrased to reflect the kind of analysis done. | We analyzed the entomological data using ANOVA and the results of the analysis indicated that there was significantly higher mosquito density on the ground floor as compared to other floors. Therefore, individuals living on the ground floor of the residential blocks are likely to be at higher risk of mosquito bites. |
